# Highly Stretchable and Flexible Melt Spun Thermoplastic Conductive Yarns for Smart Textiles

**DOI:** 10.3390/nano10122324

**Published:** 2020-11-24

**Authors:** G. M. Nazmul Islam, Stewart Collie, Muhammad Qasim, M. Azam Ali

**Affiliations:** 1 Centre for Bioengineering & Nanomedicine, Department of Food Science, Division of Sciences, University of Otago, P.O. Box 56, Dunedin 9054, New Zealand; nazmul.islam@postgrad.otago.ac.nz (G.M.N.I.); muhammadqasim.qasim@otago.ac.nz (M.Q.); 2 Bioproduct & Fiber Technology, AgResearch, Christchurch 8140, New Zealand; stewart.collie@agresearch.co.nz

**Keywords:** thermoplastic polymer, melt spinning, thermoplastic yarn, electric conductivity, wearable textile

## Abstract

This study demonstrates a scalable fabrication process for producing biodegradable, highly stretchable and wearable melt spun thermoplastic polypropylene (PP), poly(lactic) acid (PLA), and composite (PP:PLA = 50:50) conductive yarns through a dip coating process. Polydopamine (PDA) treated and poly(3,4-ethylenedioxythiophene):poly(styrenesulfonate) (PEDOT:PSS) coated conductive PP, PLA, and PP/PLA yarns generated electric conductivity of 0.75 S/cm, 0.36 S/cm and 0.67 S/cm respectively. Fourier Transform Infrared Spectroscopy (FTIR) confirmed the interactions among the functional groups of PP, PLA, PP/PLA, PDA, and PEDOT:PSS. The surface morphology of thermoplastic yarns was characterized by optical microscope and Scanning Electron Microscope (SEM). The mechanical properties of yarns were also assessed, which include tensile strength (TS), Young’s modulus and elongation at break (%). These highly stretchable and flexible conductive PP, PLA, and PP/PLA yarns showed elasticity of 667%, 121% and 315% respectively. The thermal behavior of yarns was evaluated by differential scanning calorimetry (DSC) and thermo-gravimetric analysis (TGA). Wash stability of conductive yarns was also measured. Furthermore, ageing effect was determined to predict the shelf life of the conductive yarns. We believe that these highly stretchable and flexible PEDOT:PSS coated conductive PP, PLA, and PP/PLA composite yarns fabricated by this process can be integrated into textiles for strain sensing to monitor the tiny movement of human motion.

## 1. Introduction

Smart textiles have drawn increased attention from the academic researchers and industry people due to their high sensitivity, high flexibility, breathability, multitasking capability, availability, low cost, deformability and comfort [1,2,3,4,5]. Textiles can be conductive applying various methods including spinning [6,7,8,9], knitting [10], coating [11,12,13,14,15], screen printing [16], inkjet printing [17,18] and 3D printing [19,20,21,22]. Electrically conductive yarn is one of the most basic and essential components of smart textiles due to its light weight, high stretchability, elasticity, flexibility, and comfort [23,24]. Integrating electronic mechanisms into textile structures can impart various smart functionalities including sensing, monitoring tiny body movement and information processing to conventional clothing [25].

Poly(lactic acid) (PLA) is one of the most promising and cheapest bio-based materials among the various biodegradable polyesters available in the market such as polyglycolic acid (PGA), polyhydroxybutyrate (PHB), and polycaprolactone (PCL) due to easy process characteristics [26,27]. PLA has been used in the field of biomedical, medical textiles, agricultural textiles, geo-textiles, food industry, filters, towels, home furnishings, industrial fabrics, and personal belongings due to their natural origin, adequate mechanical properties, permeability, low flammability, and excellent UV resistance [28]. Polypropylene (PP), an outstanding semi crystalline and non-polar thermoplastic polymer has been used in a wide range of applications such as protective textiles, geo-textiles, automotive interior, filaments, furniture, antistatic materials, medical devices, soft tissue replacement, plastic, piping systems, and other consumer food packaging due to its low cost, availability, gas barrier properties, adequate mechanical properties, and thermo-plasticity [29,30,31]. However, the use of PLA is restricted to the biomedical and packaging applications due to slow degradation, high processing cost and low shelf life though PLA shows high rigidity and good biocompatibility [30]. The main limitations of PLA, including low toughness and high brittleness, limit its application in stressful conditions [32,33]. It is a great challenge to achieve high levels of toughness of PLA film. The properties of PLA can be modified by blending, plasticization, and/or by reactive processing [34]. Blending polymer with other nanoparticles or polyolefin polymers is a simple method to potentially improve the property of pure polymer [35]. To minimize the above limitations of PLA, the blending of PLA with PP can lead to the desired properties such as high productivity and quick formulation changes at low price. Blending of PLA, starch, polyethylene glycol, polyethylene oxide, and polycaprolactone (PCL) with PP improves the degradability of PP. This polymer blending and composite have been widely used in biomedical textile, medical packaging, energy storage, plastic industries, and food packaging industries due to its enhanced mechanical, thermal, electrical, and biodegradation properties [36,37]. Introduction of PP with PLA decreases the stiffness property of PLA and thus enhance the mechanical properties of composite yarn. In this present investigation, melt spinning was applied to produce PP, PLA, and blend PP/PLA thermoplastic yarns due to low investment cost, solvent free simple spinning process, high production rate, and no environmental pollution. Melt spun PP, PLA, and blend PP/PLA yarns are hydrophobic and act as insulators. PP, PLA, and blend PP/PLA yarns do not absorb any chemical during coating due to hydrophobicity. Surface modifications play a vital role in a variety of application domains from electronics to medicine including interfacing with cells, bio-sensing, and drug delivery [38]. Polydopamine (PDA), a dopamine derived synthetic eumelanin polymer, can modify many kinds of substrates [12,39]. Polydopamine acts as a universal surface modification agent for different applications such as nanotechnology [40], biotechnology [41]. Here all thermoplastic yarns were treated with dopamine and Tris HCl solution. This PDA treatment converted hydrophobic yarns into hydrophilic which was proved by contact angle (CA) analysis.

Inherently conducting polymers (ICPs) such as polypyrrole, PEDOT, and polyaniline have become popular choices for producing multi-functional fibers, films, and fabrics because of their high conductivity, excellent electrochemical properties, promising catalytic activity, ease of handling, and excellent solution processability [42,43,44,45,46,47,48,49,50]. PEDOT:PSS is an automatic choice for researchers due to its high conductivity and high stability for developing highly conductive and flexible sensors for various applications such as biomedical and limb motion sensing [51], pH sensing [52,53], flexible heating element on textiles [54], strain sensing [14,55,56], temperature sensing [57], and wearable e-textiles [58]. Martin et al. [59] developed a multi-walled carbon nanotube (MWCNT, 4%)/polyethylene (PE) conducting polymeric composite by melt spinning technique and achieved conductivity only 0.1 S/cm. Soroudi et al. [60] also demonstrated filaments of blends of polypropylene (PP)/polyaniline (PANI) (20 wt%)/MWCNT (7.5 wt%) by melt spinning and this blend filaments showed maximum conductivity about 0.16 S/cm. Wang et al. [61] developed a PEDOT:PSS/PVA composite fiber via wet-spinning process for increasing the electrical conductivity and thermal stability but no information of washing is available. In this experiment, these conductive thermoplastic yarns were rinsed and the effect of rinsing on electric conductivity was assessed. These developed thermoplastic yarns also showed better electrical conductivity, thermal, and mechanical stability compared to others, which is suitable for strain sensing.

**Figure 1:**
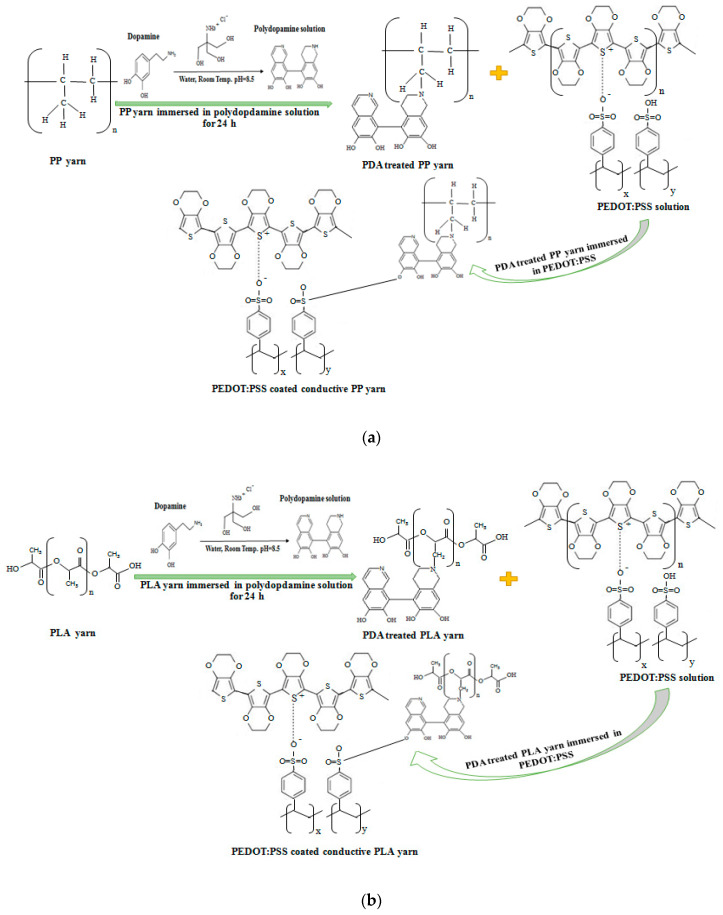
Possible key chemical reactions involved for producing PEDOT:PSS coated conductive (**a**) PP and (**b**) PLA yarn.

The target of this experiment is to fabricate PEDOT:PSS coated stable conductive thermoplastic PP, PLA and blend PP/PLA yarn which is free of metal, carbon, and silica nanoparticles. For predicting and analyzing the aging properties of conductive yarns, the aging behavior was assessed. Figure 1a,b show the key possible chemical reaction steps for producing PDA treated and PEDOT:PSS coated conductive PP and PLA yarns respectively.

## 2. Materials and Methods

### 2.1. Materials

**Figure 2:**
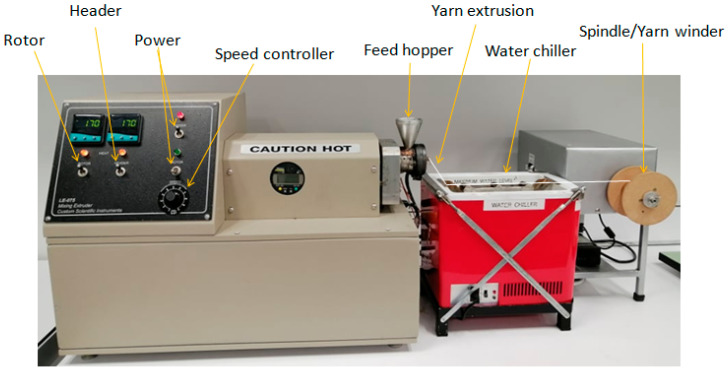
Schematic diagram of LE-075 Mixing Extruder.

A melt spinner (LE-075 Mixing Extruder, CSI, USA) shown in Figure 2 was used for producing thermoplastic melt spun yarns. Poly (lactic acid) (PLA) was provided by Imagin Plastics Ltd., Auckland, New Zealand (average molecular weight, Mw~2.08 × 10^5^, melt flow index, MFI~210 °C/2.16 kg of 15–25 g/10 min). Polypropylene (PP) was procured from Lyondell Basell, New Zealand (Mw~2.20 × 10^5^, MFI~230 °C/2.16 kg of 25 g/10 min). PEDOT:PSS dispersion was purchased from Sigma-Aldrich, New Zealand with a ratio of PSS:PEDOT = 1:1.5, pH = 2–3.5 used as conducting material. Tris hydrochloride (Tris HCl) (Bio-Froxx, GmbH, Germany) was used as buffer agent and dopamine hydrochloride (98%, Sigma-Aldrich, New Zealand) was used as binding agent for surface modification of thermoplastic yarn. Hydrochloric acid (HCl) (Sigma-Aldrich, New Zealand) was used to maintain pH = 8.5 for dopamine and Tris HCl solution.

### 2.2. Preparation of Melt Spun Conductive Composite Yarns

**Figure 3:**
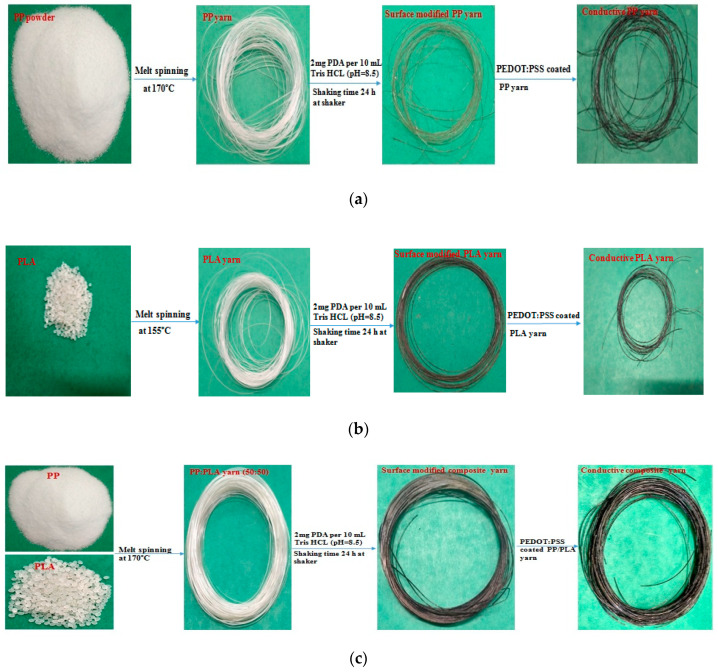
Schematic diagram of PEDOT:PSS coated conductive (**a**) PP, (**b**) PLA and (**c**) blend PP/PLA yarn.

Before thermoplastic yarn extrusion, the most important parameters such as melting point of filler and matrix, resident time, rotation per minute (rpm) for the extruder were identified. Speed/output voltage of the extruder plays a vital role for maintaining the same diameter of the fine filament. Thermoplastic yarns were produced by identifying and applying the melting point of the fillers and rpm of extrusion. In this experiment, the resident time, rpm, and speed/output voltage for the extruder were 3 min, 90 and 50 V respectively. In this present investigation, three (3) types of thermoplastic yarns were developed maintaining the residence time, voltage percentage, and rpm of extruder. Figure 3a–c illustrate the schematic diagram of PDA treated and PEDOT:PSS coated melt spun PP, PLA, and blend PP/PLA conductive yarns respectively. At first PP and PLA thermoplastic melt spun yarns were produced by a melt extruder at 170 °C and 155 °C respectively. Then a mixture (50% PP and 50% PLA) of thermoplastic polymers was manually measured and mixed and also put into the hopper of the extruder. Considering the melting point of PP and PLA, the composite thermoplastic melt spun yarn was extruded at 170 °C. For uniform blending, the produced composite yarn was cut into small sections using a scissor and again put into the feed hopper. Maintaining the same temperature, rpm, and voltage percentage of the extruder, the final composite (PP/PLA) yarn was produced by repeating this process for two times. It is mentioned that considering the residence time (3 min), rpm (90) and output voltage (50 V) of the melt extruder, the take up speed of uniform yarn production is approximately 1 m/min. The take up speed of yarn production can be increased by increasing the speed/output voltage of the melt extruder.

**Figure 4:**
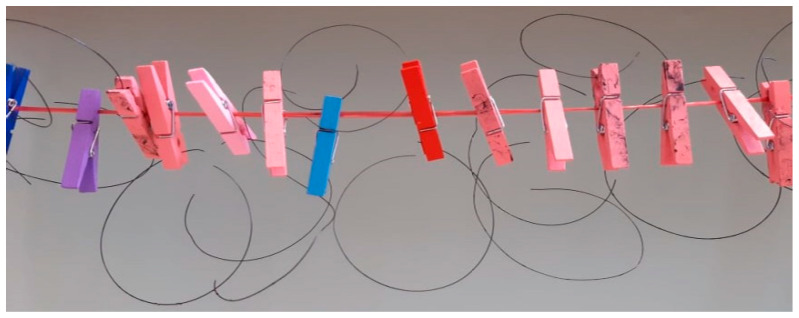
Line drying system for drying PEDOT:PSS coated conductive yarns.

These hydrophobic yarns were chemically modified to increase the hydrophilicity with PDA and Tris HCl. 12.11 g Tris HCl was added in 80 mL distilled water and pH = 8.5 was maintained by gently adding 1 µM of hydrochloric acid (HCl) for producing Tris HCl solution. 2 mg of dopamine hydrochloride per 10 mM Tris HCl was added to produce the aqueous solution. PP, PLA, and blend PP/PLA based hydrophobic yarns were immersed in this aqueous solution and kept in a shaker for 24 h with 55 rpm at room temperature. The surface modified yarns were rinsed with distilled water for 1 min and line dried at room temperature. These PDA treated hydrophilic PP, PLA and blend PP/PLA yarns were immersed in PEDOT:PSS dispersion for 5 min. Then the coated yarns were dried at room temperature for 4 h by hanging them on a clothes line using wooden clip hangers shown in Figure 4. This coating process was repeated for two dip coating cycles. All the thermoplastic yarns were coated for two times considering the flexibility, stiffness, and rigidity of conductive yarns. Though more coating cycles increased the conductivity but made the yarns stiff and rigid. Stiff and rigid yarns are not suitable for integrating into textiles for wearable applications. The above fabrication process can be described as: production of thermoplastic yarns by melt spinning > surface modification by polydopamine > PEDOT:PSS coated conductive yarns by dip coating.

### 2.3. Characterizations of Thermoplastic Yarns

The chemical interactions among different components such as pure PP, pure PLA, blend PP/PLA, PDA, and PEDOT:PSS were studied using FTIR. This measurement was performed with a total of 24 scans/sample over the range of 4000–400 cm^−1^ at resolution of 4 cm^−1^.

The contact angle (CA) was analyzed using FTA200 Dynamic Contact Angle Analyzer (First Ten Angstroms, Portsmouth, VA, USA) with the static sessile drop method. A droplet (1 μL) of distilled water was placed on the surface of the raw thermoplastic and PDA treated yarns by a syringe. A video camera (Sony ICX274 CCD) was used to record the water contact angles of the raw thermoplastic and PDA treated yarns at room temperature.

The DC electrical resistance of 20 cm lengths of conductive yarns was measured by FLUKE 114 TRUE RMS Multimeter (Everett, WA, USA) before and after rinsing. Electric resistance was measured three times for each conductive yarn every after each dip coating cycle and averaged. Then electric conductivity (σ) was measured applying the following formula [62]:

σ = L/RA(1)

where R is the electrical resistance (Ω), A is the total cross-sectional surface area (cm^2^) and L is the distance between electrodes (cm).

Optical microscopy was performed on several pure PP, pure PLA, blend PP/PLA, surface modified, and PEDOT:PSS coated yarns to determine the presence of PDA and conductive polymer on pure yarns. Each yarn was attached at both sides on a glass slide using clear scotch tape exposing 30 mm of yarn. Optical microscopy was carried out by an optical microscope (OLYMPUS, Tokyo, Japan). Optical images were captured with a HUWAEI Y9 camera (Shenzhen, China) and images were cropped using Photoshop software. An image of the all yarns at 100 times magnification was captured in all stages in the same position.

For analyzing the yarn surface morphology, PDA treated and PEDOT:PSS coated samples were attached to the scanning electron microscope (SEM) specimen stub using double sided carbon tape. Before SEM analysis, they were sputter-coated with 5 nm gold-palladium using a Q150T sputter coater (Quorum Technologies Ltd., East Sussex, UK) in order to prevent the surface charging effect which gives a blurred picture and to promote the emission of secondary electrons for providing a homogeneous surface for analysis. This morphological analysis of yarns was characterized using Tabletop Microscope TM3030 (Hitachi, Japan) with voltage of 15 kV at different magnifications. The thickness of the coating was measured using ImageJ software by taking three measurements of six different samples of the coatings. Data are expressed as mean ± SD.

The washing stability of conductive yarns of each dip cycle was assessed. The coated conductive yarns of each dip cycle were rinsed for 1 min and line dried for 2 h at room temperature. Then their electrical resistance was measured. This rinsing process was carried out five times.

Differential Scanning Calorimetry (DSC) was performed by TA analyzer (TA) Q1000 instrument (TA Instruments, New Castle, DE, USA) to measure the glass transition (T_g_) and melting (T_m_) temperature characteristics of thermoplastic PP, PLA, and blend PP/PLA conductive yarns to determine the thermal stability. Samples were weighed (10–15 mg) into a pan (Tzero pan; TA Instruments Ltd., New Castle, DE, USA). Each sample was heated over the temperature range from 20 to 200 °C at the rate of 5 °C/min under nitrogen atmosphere (50 mL/min).

The thermal stability and degradation of pure PP, pure PLA, PP/PLA, PDA treated and PEDOT:PSS coated yarns were analyzed by Q50 TGA analyzer (TA instruments, New Castle, DE, USA). The weight of samples was 20–35 mg. These stability analyses were performed over the temperature range 200 to 600 °C at a heating rate 20 °C/min under the nitrogen atmosphere (20 mL/min).

The mechanical properties of all yarns were investigated by a TA.HD plusC Texture Analyzer (UK) applying 5 kg load cell, gauge length of 25 mm and tensile speed 20 mm/min at room temperature.

To evaluate the aging effect, the conductive yarns of each dipping cycle were stored in a ambient room conditions for five weeks in separate polythene bags with minimal exposure to air and moisture. The loss of electrical resistance during aging was measured every week in order to determine the shelf life of conductive yarn.

## 3. Results and Discussion

### 3.1. Fourier Transform Infrared Spectroscopy (FTIR) Analysis

**Figure 5:**
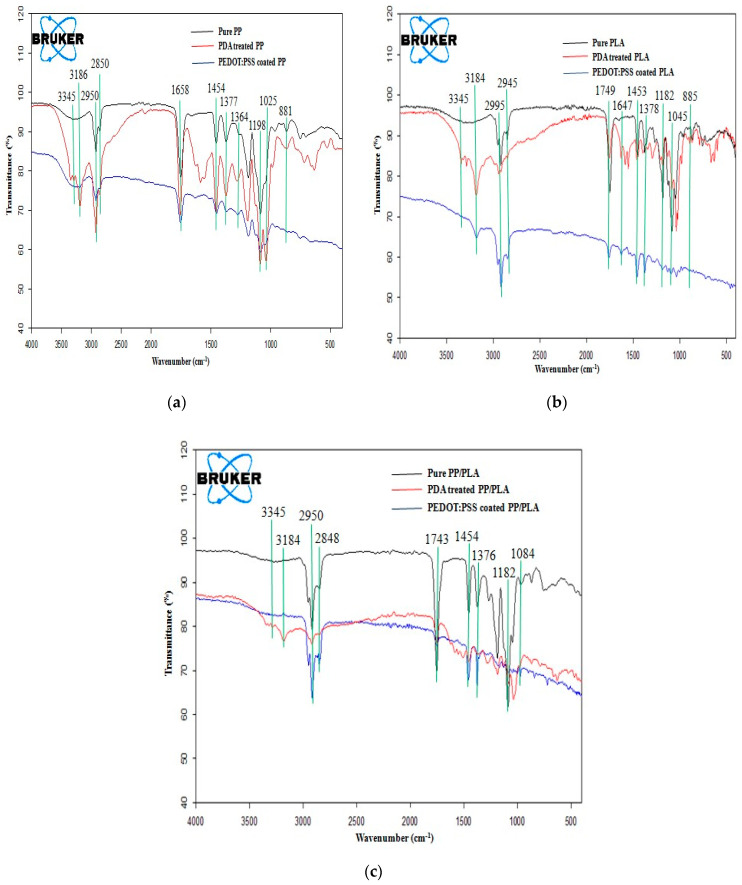
FTIR spectra of PEDOT:PSS coated (**a**) PP, (**b**) PLA and (**c**) blend PP/PLA yarn.

The functional groups of all the samples such as pure PP, pure PLA, blend PP/PLA, PDA and PEDOT:PSS were confirmed by interpretation of the FTIR spectra. Figure 5a, Figure 5b, and Figure 5c represent the FTIR spectra of PDA treated and PEDOT:PSS coated conductive PP, PLA and PP/PLA yarns respectively. All the transmittance bands are also listed in Table 1. Here Figure 5a depicts the transmittance bands corresponding to PP at 2950–2850 cm^−1^, 1454 cm^−1^ and 1377 cm^−1^ were assigned to C–H stretching, –CH_3_ bending and C–H bending respectively [30]. Here Figure 5b depicts the FTIR spectra of PLA, transmittance bands at 2995–2945 cm^−1^, 1749 cm^−1^, 1182–1045 cm^−1^ and 1453 cm^−1^ referred to CH and CH_3_ group, C=O stretching, symmetric C–O–C stretching and asymmetric bending absorption of CH_3_ respectively [63]. Ploypeetchara et al. [64] analyzed the spectra of different PP/PLA ratios and found the transmittance bands that represent PP and PLA were observed in the PP/PLA blend around 2952–2848cm^−1^, 1456–1454 cm^−1^, 1376 cm^−1^, 1183–1182 cm^−1^ and 1086–1184 cm^−1^. A specific peak for all PP/PLA blends appeared at 1749 cm^−1^ is corresponded to the stretching of the ester group (–COO) where the chemical interaction of the anhydride group of PP with the carbonyl group of PLA formed a new linkage which indicates the PP/PLA blends [37]. From Figure 5c, the transmittance bands that represent PP and PLA were observed in the PP/PLA blends around 2950–2848 cm^−1^, 1743 cm^−1^, 1454 cm^−1^, 1376 cm^−1^ and 1182 cm^−1^. After surface modification of PP, PLA and PP/PLA by PDA, the transmittance bands at 3186–3345 cm^−1^; 3184–3345 cm^−1^ and 3184–3345 cm^−1^ respectively corresponded to stretching vibrations of O–H and N–H groups of PDA [65].

From Figure 5a–c, it is seen that the PDA was coated successfully onto the surfaces of PP, PLA and PP/PLA yarns. From Figure 5a it is seen that the absorption spectra of PEDOT:PSS coating on PDA treated conductive polypropylene yarn displayed the polymeric interactions in the thiophene backbone including C=C, C–C and C–S bonds at 1658 cm^−1^, 1364 cm^−1^, 1198 cm^−1^ and 1025–881 cm^−1^ respectively [63]. Similarly Figure 5b shows that the FTIR spectra of PEDOT:PSS coated and PDA treated conductive PLA yarn displayed the polymeric interactions in the thiophene back bone, including C=C, C–C and C–S bonds at 1647 cm^−1^, 1378 cm^−1^, 1199 cm^−1^ and 1025–885 cm^−1^ respectively [63]. Figure 5c also indicates that the absorption spectra of PDA treated and PEDOT:PSS coated conductive PP/PLA yarn displayed the transmittance bands at 1647 cm^−1^, 1376 cm^−1^, 1183 cm^−1^ and 1084–1042 cm^−1^ corresponded to C=C, C–C and C–S bonds respectively [66]. From Figure 5a–c, it is confirmed that after two coating layers of PEDOT:PSS on PDA treated PP, PLA, and PP/PLA yarns, all transmittance bands were found to be almost similar due to low PSS adsorption.

### 3.2. Contact Angle (CA) Analysis

**Figure 6:**
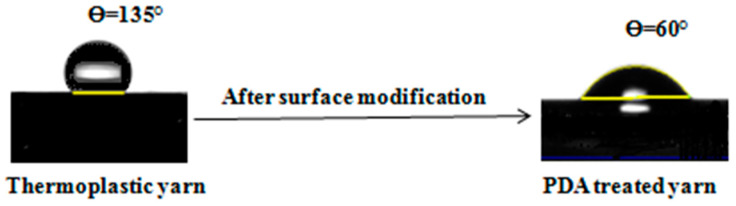
The images of water contact angle of untreated and PDA treated thermoplastic yarns.

Wettability, an important phenomena of substrates which is related to the surface roughness and surface charge. Here the melt spun thermoplastic yarns do not absorb any chemicals due to their hydrophobicity. Using the general method of contact angle measurement, it is hard to analyze a tiny fiber and yarn. Therefore, we modified the procedure and used adhesive tape to put yarn on contact angle machine stage. For dropping water on yarn surface, we did not use the machine connected syringe pump. However, we manually placed the drop of water on yarn surface using micro-pipette volume (1 µL). The contact angles (ϴ) of raw thermoplastic polypropylene yarn and PDA treated polypropylene yarn were measured and shown in Figure 6. From Figure 6, it is seen that the contact angle (CA) of raw thermoplastic polypropylene yarn is ϴ = 135°. It has a CA value of 135° in all groups before surface modification. As this raw thermoplastic polypropylene yarn is hydrophobic, the surface of this yarn was modified by polydopamine. After polydopamine treatment, the CA of treated polypropylene yarn decreased which is ϴ = 60°. From the CA of PDA treated yarn, it is confirmed that PDA converted hydrophobic thermoplastic polypropylene yarns into hydrophilic.

### 3.3. Electrical Conductivity before Rinsing

Electrical conductivity is one of the most important key aspect and requirements for wearable conductive yarns. Table 2 shows the electric conductivity of PDA treated and PEDOT:PSS coated 20 cm long conductive PP, PLA, and blend PP/PLA yarns before rinsing.

After the first and second dip coating cycles, the electric conductivity of conductive PP yarn is 0.25 S/cm and 0.75 S/cm, respectively. Similarly, the electric conductivity of conductive PLA yarn is 0.17 S/cm and 0.36 S/cm respectively. In addition, the electrical conductivity of blend PP/PLA yarn is 0.24 and 0.67 S/cm respectively before rinsing. The number of dip coating cycle increases the electrical conductivity of the coated yarns. After the second coating cycles, the electrical conductivity of each yarn increases at least two times compared to the first coating cycle. In Table 2, it is seen that the PDA treatment has converted the hydrophobic yarns into hydrophilic yarns successfully which was proved by contact angle analysis and the number of coating cycles increased the PEDOT:PSS pick up% which increased the electrical conductivity.

### 3.4. Tensile Properties Analysis

**Figure 7:**
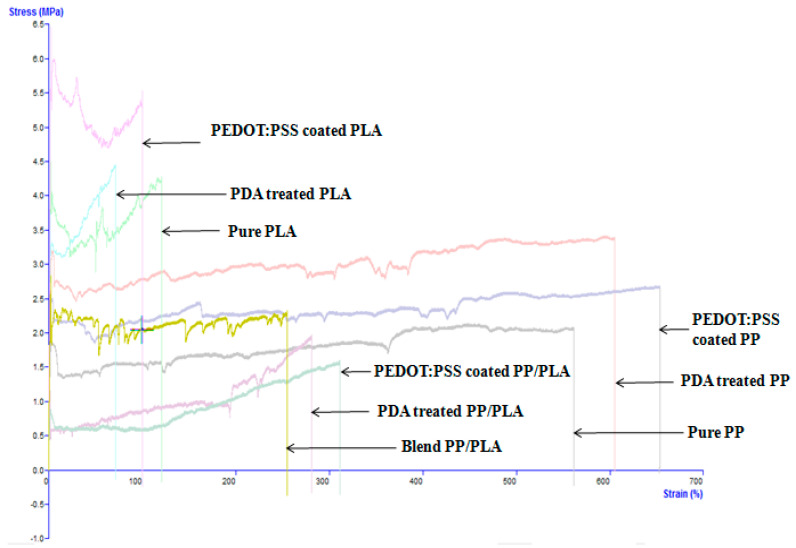
The stress-strain curves of various thermoplastic yarns.

The mechanical properties (tensile strength, Young’s modulus, and elongation at break %) of pure PP, pure PLA, pure PP/PLA, PDA treated and PEDOT:PSS coated conductive PP, PLA, and PP/PLA yarns were investigated. The role of the PDA introduction and PEDOT:PSS coating on melt spun PP, PLA and PP/PLA yarns was characterized by their mechanical properties. Figure 7 illustrates the stress-strain curves to analyze the mechanical properties of the various types of yarns. Three (3) replicates were tested for each yarn and the average values of tensile strength, Young’s modulus and elongation at break (%) were reported in Table 3.

Figure 7 shows all full stress-strain curves for analyzing the tensile strength, Young’s modulus, and elongation at break%. From Table 3, it is seen that PLA yarns have better tensile strength compared to PP and PP/PLA yarns. The tensile strength of pure PP, pure PLA, blend PP/PLA, polydopamine treated and PEDOT:PSS coated PP, PLA and PP/PLA is 1.22 MPa, 1.81 MPa, 1.97 MPa; 2.99 MPa, 3.41 MPa, 3.57 MPa; and 1.35 MPa, 2.08 MPa, 2.56 MPa respectively. Moreover, the tensile strength of PP/PLA blends improved with the addition of the PLA content due to the higher Young’s modulus of the PLA yarn compared to the PP yarn. The bridged two immiscible PP and PLA polymers have formed a strong chemical bond which was confirmed by the FTIR analysis. However, polydopamine treatment and PEDOT:PSS coating also increased the mechanical properties of these treated and coated yarns. The mechanical properties of PP/PLA blends are strongly influenced with greater physical properties of PLA including the degree of crystallinity, melting point, density, heat capacity hardness, Young’s modulus, tensile strength, glass transition temperature, and mechanical properties.

From Table 3 it is seen that the Young’s modulus of pure PP, pure PLA, blend PP/PLA, polydopamine treated and PEDOT:PSS coated PP, PLA and PP/PLA is 76.98 MPa, 87.92 MPa, 116.39 MPa; 230.70 MPa, 291.87 MPa, 309.29 MPa; 96.16 MPa, 172.11 MPa and 188.40 MPa respectively. The elongation at break of pure PP, pure PLA, blend PP/PLA, polydopamine treated and PEDOT:PSS coated PP, PLA and PP/PLA is 594.53%, 636.51%, 667.47%; 42.30%, 76.89%, 121.35% and 227%, 264%, 315% respectively. So it is obvious that the polydopamine treatment and PEDOT:PSS coating have played vital a role for improving the mechanical properties of blend PP/PLA yarn.

However, it is clearly exhibited that introducing of PDA treatment and PEDOT:PSS coating illustrated a good improvement of mechanical properties of the treated and coated PP, PLA and PP/PLA yarns due to the -NH_2_ functional group of dopamine and C-S bonds reaction happened among PDA treated thermoplastic yarns and PEDOT:PSS. This developed conductive yarns showed higher elongation at break% compared to others development. For example, Luo et al. [67] developed PEDOT:PSS/PDMS blend conductive polymer films which showed elongation at break of about 82%. Azizi et al. [37] also developed PP, PLA, and PP/PLA nanocomposite and the elongation at break of PP, PLA and PP/PLA are 210%, 20% and 25–150% respectively. So this high stretchability feature from this present investigation was a good upshot for this study which may also be an intelligent aspects of these new yards to be applied for strain sensing application.

### 3.5. Optical Microscopy Images Analysis

**Figure 8:**
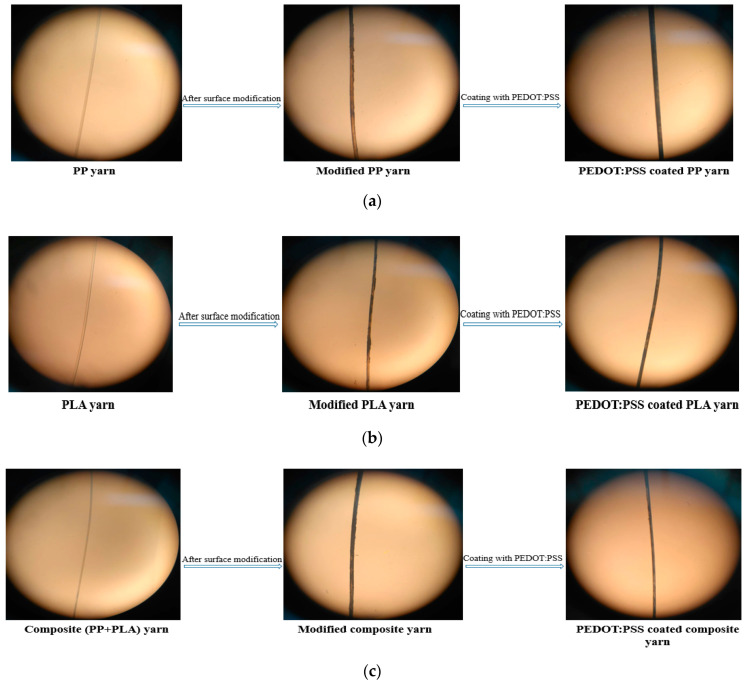
Optical microscopic images of conductive (**a**) PP, (**b**) PLA and (**c**) blend PP/PLA yarn.

Optical microscopy images were used to analyze the coating thickness of the PP, PLA and PP/PLA yarns at different stages to analyze the changes of coating thickness of PP, PLA and PP/PLA yarns after PDA treatment and PEDOT:PSS coating. Optical microscope images captured of several single yarns in a original state before PDA treatment, after PDA treatment and PEDOT:PSS coating show that there are significant changes in the thickness of boundary layers shown in Figure 8. It is mentioned that an image of the all yarns at 100 times magnification was captured in all stages.

From Figure 8, it is seen that PDA treatment converts the white color of pure PP, PLA, and blend PP/PLA yarn into black which confirms the successful coating on thermoplastic yarns. So it can be assumed that PDA treatment has a great impact in the increased thickness of boundary layers of yarns and PEDOT:PSS pickup%.

### 3.6. Scanning Electron Microscope (SEM) Analysis

**Figure 9:**
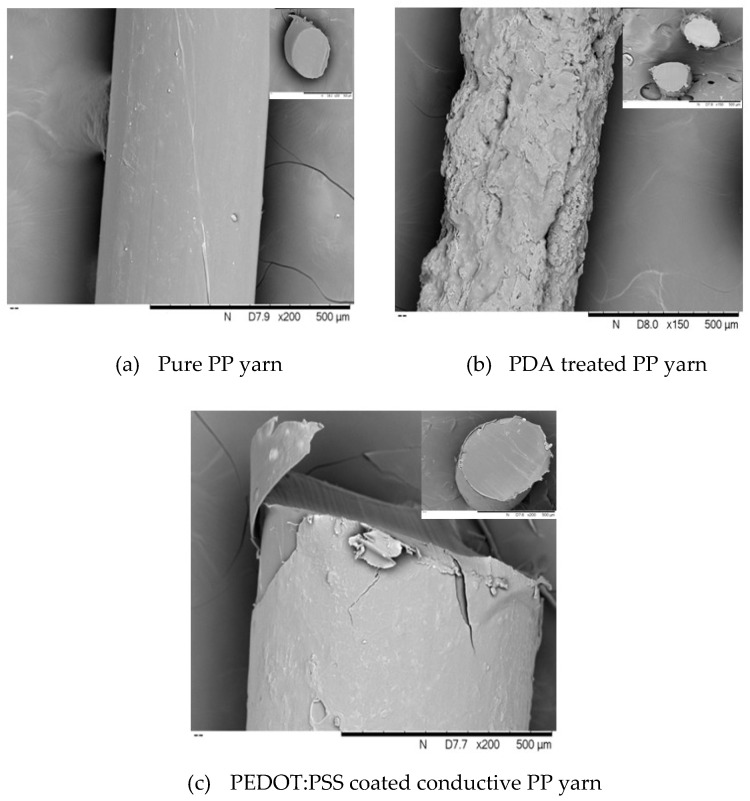
SEM images (surface and cross-section) of PP yarn at various stages.

**Figure 10:**
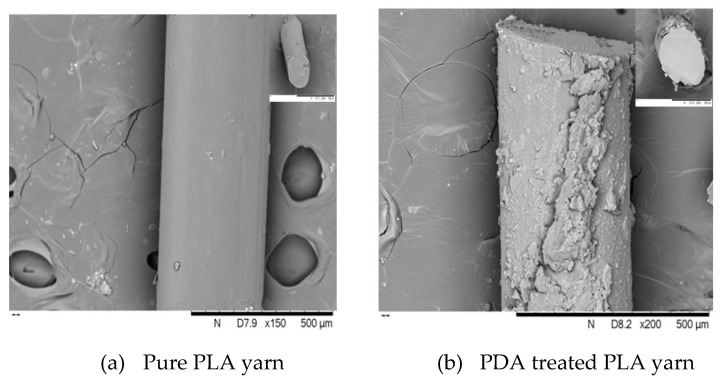

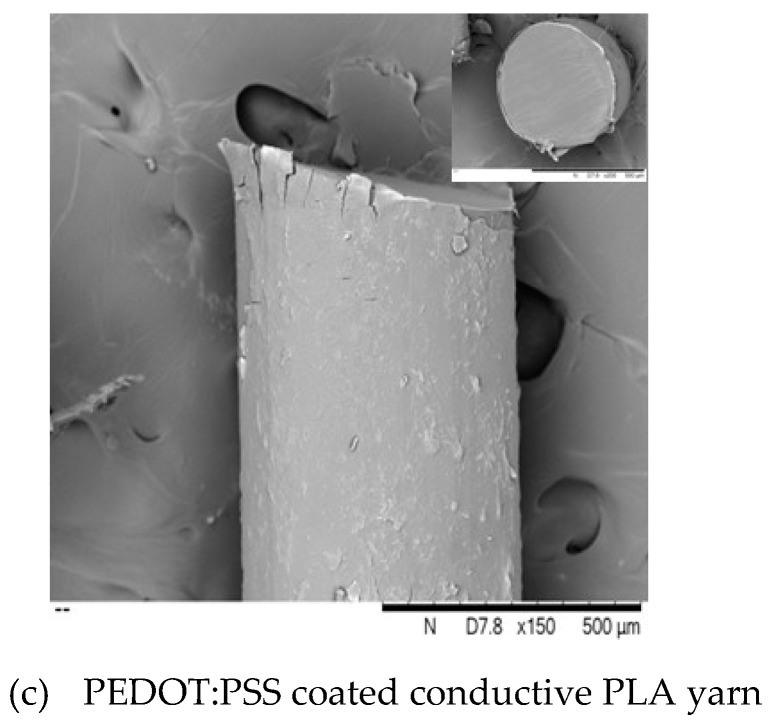
SEM images (surface and cross-section) of PLA yarn at various stages.

**Figure 11:**
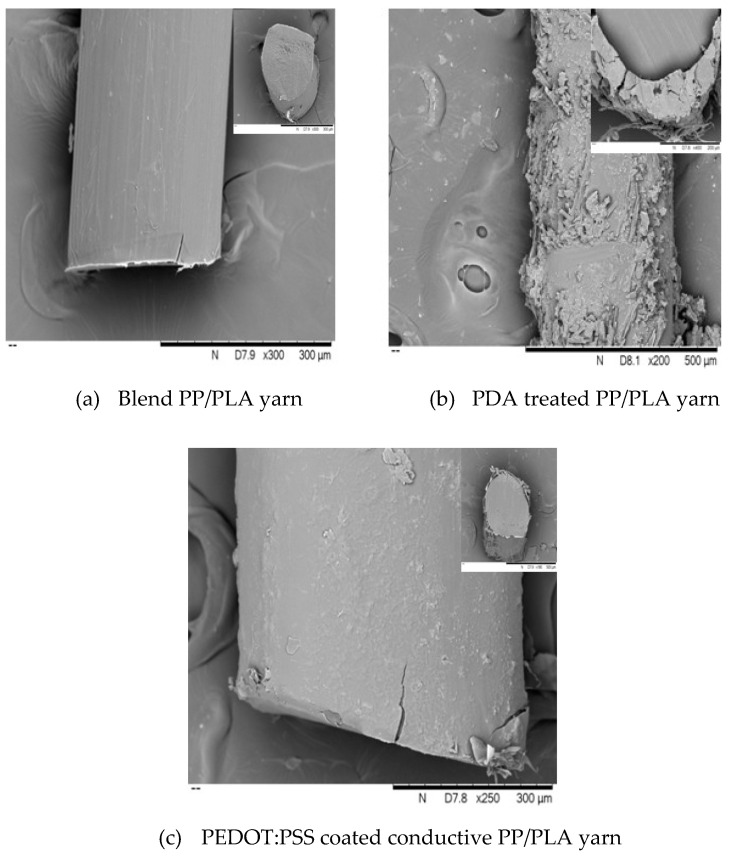
SEM images (surface and cross-section) of PP/PLA yarn at various stages.

The surface morphology of pure PP, pure PLA, blend PP/PLA, PDA treated, and PEDOT:PSS coated conductive PP, PLA, and PP/PLA yarns were also analyzed using SEM as shown in Figure 9, Figure 10, and Figure 11 respectively. A smooth surface morphology was observed without PDA coating while rough surface was observed in yarns which had been coated with PDA. This fact is evident from Figure 9a, Figure 10a and Figure 11a as a flat, smooth, and featureless surface of pure PP, pure PLA, and blend PP/PLA yarn can be observed.

Figure 9b, Figure 10b, and Figure 11b revealed densely rough, more intact features and granular morphology of PDA treated PP, PLA and PP/PLA yarns. After surface modification of thermoplastic yarns by PDA, the results showed that the thickness of the modified PP, PLA and PP/PLA coating were 3.96 ± 1.45 µm, 3.52 ± 5.12 µm and 6.71 ± 3.9 µm respectively. Though PDA coating layer was observed over surfaces of PP, PLA and PP/PLA surface but it was not smooth and cracks are visible. However, still this roughness created a hydrophilic base for further coating and increased the tensile strength of yarns.

Figure 9c, Figure 10c, and Figure 11c displayed the PEDOT:PSS coated conductive PP, PLA and PP/PLA yarns respectively. A clear wrapping of PEDOT:PSS can be seen in Figure 9c, Figure 10c and Figure 11c over the yarns of PP, PLA, and blend PP/PLA. After PEDOT:PSS coating, the thickness of coating layer of the coated PP, PLA and PP/PLA were 5.79 ± 1.44 µm, 5.62 ± 1.0 µm and 8.3 ± 2.3 µm respectively. This smooth morphology is critical for conductivity of materials as brittle surface can act as a barrier to flow of charges. The possible reason of achieving smooth surfaces of PEDOT:PSS coated thermoplastic yarns may be attributed to higher wettability, absorption and better linkage between the conductive polymer dispersion and the flexible substrates [68].

### 3.7. Thermal Behavior Analysis

#### 3.7.1. Thermo-Gravimetric Analysis (TGA)

**Figure 12:**
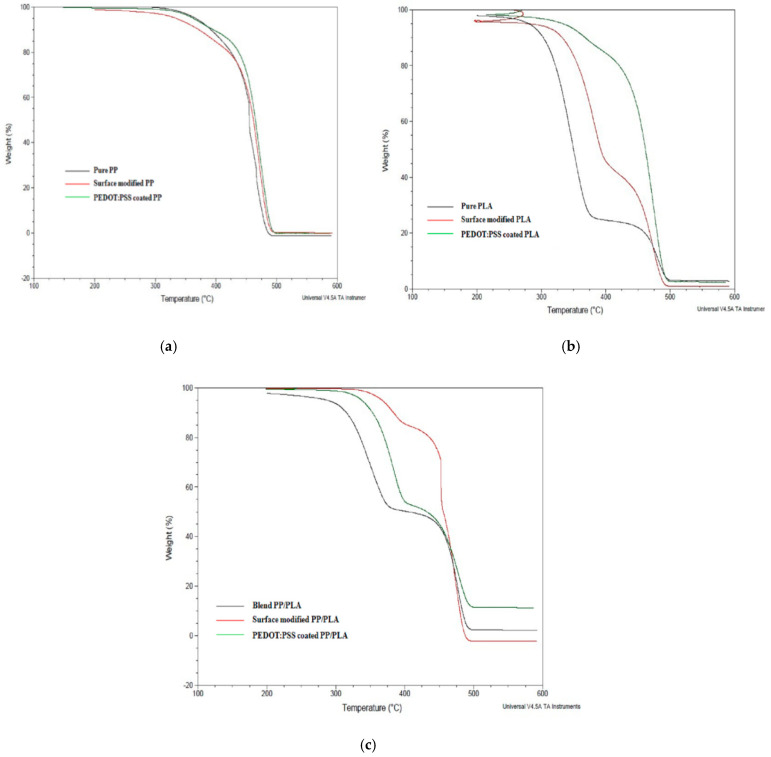
Thermogravimetric analysis (TGA) of PDA treated and PEDOT:PSS coated (**a**) PP, (**b**) PLA and (**c**) PP/PLA yarn.

The thermal stability of pure PP, pure PLA, blend PP/PLA, PDA modified and PEDOT:PSS polymer coated conductive yarns were analyzed by thermos-gravimetric analysis under nitrogen atmosphere are shown in Figure 12a–c respectively. Here 5% and 50% mass loss occurring was investigated to maintain the accuracy of the thermal degradation temperatures characteristics. The two degradation temperatures T_5%_ and T_50%_ correspond to 5% and 50% mass loss of the samples respectively. The remaining ash (%) at 500 °C was also measured to determine the stability of various yarns. The mass loss (5%, 50%) and the remaining ash (%) were summarized in Table 4.

The thermal degradation curves of pure PP and pure PLA are also shown for comparison with modified and coated yarns. Figure 12a,b illustrate that the pure PP, PLA, modified and coated yarns experience single stage mass loss. Remaining ash (%) at 500 °C indicates that the introduction of PDA treatment and PEDOT:PSS coating improve the thermal stability of polymers with increase onset thermal degradation temperature and high molecular chain interaction with thermoplastic polymer.

From Figure 12c, it is seen that the thermo-grams of blend PP/PLA polymers reveal two-step degradation processes which indicate two mass loss. The first weight loss is due to the vanishing of the ester groups in the PLA polymer structure [37]. The second weight loss observed at ~380 °C which indicates the decomposition of PP polymer. The addition of PLA in PP polymer to produce blend PP/PLA decreases the initial degradation temperature to 315 °C due to the incompatibility between PP and PLA polymers. However, the introduction of PDA and PEDOT:PSS coating increase the interfacial adhesion between PP and PLA. From remaining ash (%) at 500 °C, it is confirmed that the thermal stability of thermoplastic yarns has been enhanced by addition of PDA and PEDOT:PSS coating.

#### 3.7.2. Differential Scanning Calorimetry (DSC) Analysis

**Figure 13:**
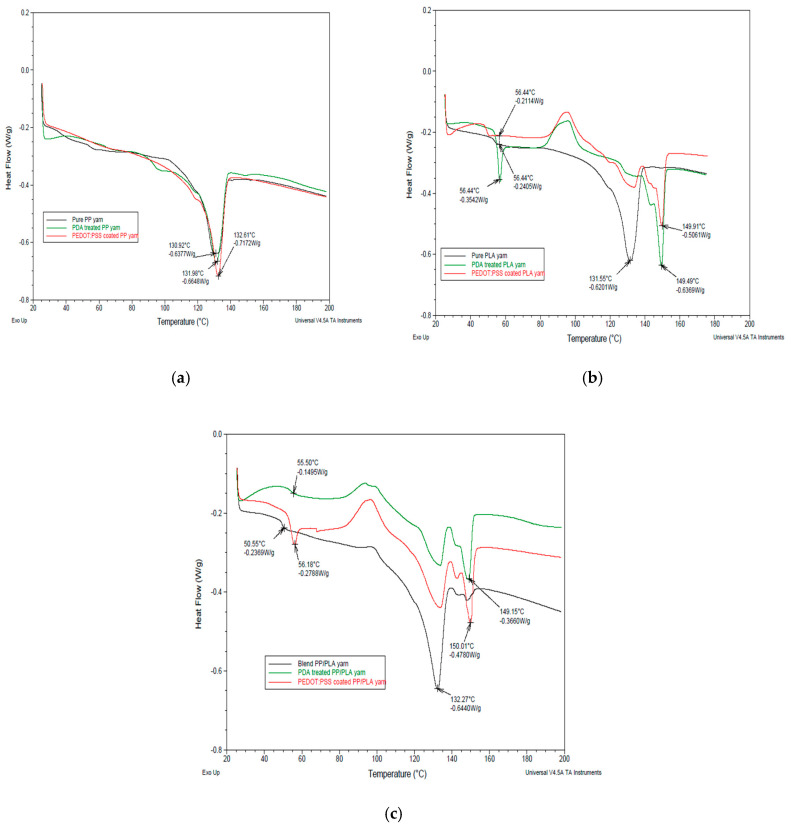
Differential scanning calorimetry (DSC) thermograms of PDA treated and PEDOT:PSS coated (**a**) PP, (**b**) PLA and (**c**) blend PP/PLA yarn.

To determine the thermal properties of pure PP, pure PLA, blend PP/PLA, PDA treated and PEDOT:PSS coated conductive yarns, DSC analysis was carried out and the thermo-grams are shown in Figure 13a–c respectively. The glass transition temperature (T_g_) and melting temperature (T_m_) are summarized in Table 5. From Figure 13a, it is seen that no glass transition temperature is detected. The melting temperature (Tm) of PP yarn is detected at 130.92 °C [69]. After PDA treatment and PEDOT:PSS coating, melting point temperature (T_m_) of PP is revealed at 131.98 °C and 132.61 °C respectively. So it is noted that PDA treatment and PEDOT:PSS coating have increased the melting point (T_m_) of PP polymer. Moreover, from Figure 13b, it is seen that the T_g_ of the pure PLA shows a hysteresis peak. The T_g_ and T_m_ of PLA are 56.44 °C [64] and 131.55 °C [70] respectively.

The T_g_ of the PLA remains same due to the reduction of the mobility of the amorphous character in the PLA polymer and the physical cross links with lower addition of PDA and PEDOT:PSS. A considerable increase is found in the T_m_ of PLA after PDA treatment and coating due to the physical crosslink with PLA polymer. From Figure 13c, it is seen that the glass transition temperature of blend PP/PLA polymer is 50.55 °C [64]. After polydopamine treatment and PEDOT:PSS coating, the glass transition temperature of blend PP/PLA polymers have increased at 55.50 °C and 56.18 °C respectively.

Polymer blending of PLA with PP decrease the melting point of PP. The melting point of blend PP/PLA is 132.27 °C. The melting points of polydopamine treated and PEDOT:PSS coated blend PP/PLA yarn are 149.15 °C and 150.01 °C respectively. So it is obvious that the addition of PEDOT:PSS coating increases the melting point of blend PP/PLA polymer because of the increased interfacial adhesion and interaction between the two polymer chains.

### 3.8. Aging Effect on Electrical Conductivity under Different Processing Conditions

For predicting and improving the shelf life of the developed conductive yarn, it is essential to analyze the degradation of the conducting material under end-use conditions during aging. Textile sensors will be used in several times. Consumers will wear this type of sensors and go outside. Then this sensors will be exposed to moisture, oxygen, and sunlight. So the developed conductive yarns were stored in a real conditioning room maintaining the parameters (temp. 20 °C and R.H. 65 ± 4%) for five weeks to analyze and measure the effect of oxygen, sunlight, and moisture content on the electric conductivity of conductive yarns. Table 6 represents the aging effect on electrical resistance of PEDOT:PSS coated conductive PP, PLA and PP/PLA yarn.

Here the gain of electrical resistance on aging was evaluated in every week. By calculating this total increased electrical resistance (%), the effect of atmospheric storage was analyzed. Considering the dip coating cycle 1 and 2, electrical resistance of conductive PP, PLA and PP/PLA yarn increased by ~23.43%, ~20.98%; ~26.85%, ~23% and ~25.45%, ~21.96% respectively in five weeks due to aging under storage conditions. Considering the increase of electric resistance, it is confirmed that aging has enormous effect on the shelf life of these conductive materials. The reason for increase in the electrical resistance of PEDOT:PSS coated conductive yarns may be the oxidative degradation by oxygen and the degradation of the conductive material by the atmospheric moisture.

### 3.9. Electrical Conductivity after Rinsing

**Figure 14:**
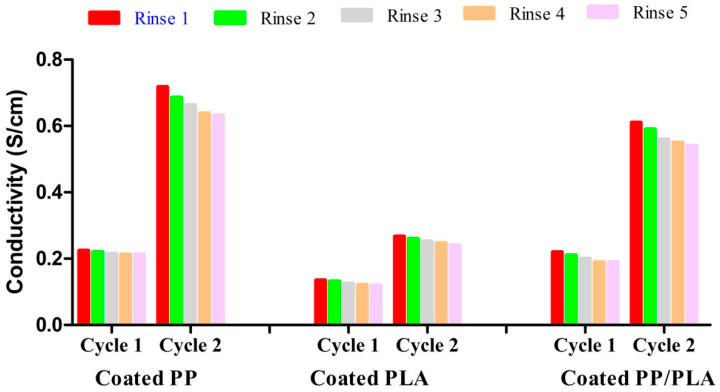
Conductivity (S/cm) of PEDOT:PSS coated PP, PLA and PP/PLA yarns after rinsing.

Conductive yarns must be sufficiently robust to be suitable for daily use particularly in respect of bending, abrasion, and cleaning. Wash durable conductive yarns production is a great technical challenge for repeat use. Conductive tracks typically cannot survive machine washing due to the mechanical stresses, reaction between detergent and water. The cleaning stability and washing performance of both PEDOT:PSS coated conductive yarns were analyzed. Figure 14 shows the relationship between electrical conductivity (S/cm) and rinsing cycles of PEDOT:PSS coated PP, PLA, and PP/PLA yarns after five rinsing cycles. Electrical conductivity of the conductive yarns was considerably decreased every after rinsing cycle.

Before rinsing the conductivity for dip coating cycle 1 and cycle 2 of conductive PP yarn was 0.25 S/cm and 0.75 S/cm respectively. After 5th rinsing of conductive PP yarn, the conductivity for dip coating cycle 1 and cycle 2 is 0.213 S/cm and 0.632 S/cm respectively. The decreased conductivity of PP yarn for cycle 1 and cycle 2 is 14.8% and 15.73% respectively. Similarly before rinsing the conductivity for dip coating cycle 1 and cycle 2 of conductive PLA yarn was 0.17 S/cm and 0.36 S/cm respectively. After 5th rinsing of conductive PLA yarn, the conductivity for dip coating cycle 1 and cycle 2 is 0.12 S/cm and 0.241 S/cm, respectively. Therefore the decreased conductivity of PLA yarn for dip coating cycle 1 and cycle 2 is 29.41% and 33.06% respectively. Before rinsing the conductivity for dip coating cycle 1 and cycle 2 of conductive composite (PP/PLA)yarn was 0.24 S/cm and 0.67 S/cm respectively. After 5th rinsing of conductive PP/PLA yarn, the conductivity for dip coating cycle 1 and cycle 2 of conductive PP/PLA yarn is 0.19 S/cm and 0.54 S/cm respectively. The decreased conductivity of blend PP/PLA yarn for dip coating cycle 1 and cycle 2 is 20.83% and 19.40%, respectively. This decreased electrical conductivity could be due to the removal of excess unfix PEDOT:PSS on the yarn surface. Here conductive PP yarn showed better cleaning stability compared to conductive PLA and composite PP/PLA yarn.

## 4. Conclusions

A new class of smart interactive textiles (*i*-textiles) is being designed to develop new strategies toward smart materials for innovative applications in the various fields including public safety, healthcare, artificial muscles, military, strain sensing, space exploration, stretchable displays, sports, and consumer fitness. This manuscript detailing of it study results has demonstrated to construct highly stretchable, cost effective, durable, and environmentally friendly melt spun thermoplastic conductive yarns with excellent thermal and mechanical properties. Here we have introduced mussel-inspired polydodapine (PDA) treatment to modify the surface of the melt spun thermoplastic yarns. This PDA treatment acts not only as a coupling or bonding agent but also as plasticizer. This dual characteristics illustrate significant improvement of surface properties of the thermoplastic yarns. These PDA treated thermoplastic yarns consisting of PP, PLA, and PP/PLA that were effectively coated with PEDOT:PSS toward increasing the efficacy of wearable textile sensors. Mechanically the conductive PP, PLA, and PP/PLA yarns were highly stretchable and flexible. These highly stretchable and flexible conductive yarns can be used for producing conductive textiles by knitting and also can be integrated into any textile substrates/fabrics by sewing. The usage of these conductive yarns might be applied to analyze sporting performance and heart beat of a sportsman, tiny joint movement of human body, the health record of patients, speaking, swallowing and breathing.

## Figures and Tables

**Table 1:** FTIR transmittance bonds of thermoplastic yarns.

IR Absorption Bands (cm^−1^)	Description
3184–3345	stretching vibrations of O–H and N–H groups
2945–2850	C–H stretching
1743–1454	–CH_3_ stretching
1647–1658	C=C stretching
1045–1182	C=O stretching
1376	C–H bending
1183 and 1025–881	Stretching C–S
1453	Symmetric C–O–C stretching

**Table 2:** Electrical conductivity (S/cm) of PEDOT:PSS coated PP, PLA, and blend PP/PLA yarn before rinsing.

Yarn Type	Radius (cm)	Area (cm^2^)	Coating Cycle 1			Coating Cycle 2		
			Mean Electrical Resistance (kΩ)	SD	Conductivity (S/cm)	Mean Electrical Resistance (kΩ)	SD	Conductivity (S/cm)
PP	0.014	0.000616	131.00	3.42	0.25	43.04	2.20	0.75
PLA	0.013	0.000531	223.33	7.77	0.17	122.67	5.47	0.36
PP/PLA	0.012	0.000452	181.13	3.62	0.24	65.93	1.23	0.67

SD = Standard Deviation.

**Table 3:** Mechanical properties of thermoplastic yarns.

Types of Yarn	Tensile Strength (MPa)	SD (MPa)	Tensile/Young’s Modulus (MPa)	SD (MPa)	Elongation at Break (%)	SD
PP Modified PP PEDOT:PSS coated PP	1.22 1.81 1.97	0.14 0.09 0.02	76.98 87.92 116.39	4.99 5.47 8.94	594.53 636.51 667.47	5.76 7.98 5.92
PLA Modified PLA PEDOT:PSS coated PLA	2.99 3.41 3.57	0.18 0.40 0.23	230.70 291.87 309.29	7.67 5.78 7.51	42.30 76.89 121.35	0.39 0.63 0.48
PP/PLA Modified PP/PLA PEDOT:PSS coated PP/PLA	1.35 2.08 2.56	0.15 0.49 0.08	96.164 172.11 188.40	7.71 5.33 5.76	227.17 263.64 315.33	1.34 1.01 0.98

SD = Standard Deviation.

**Table 4:** TGA results of conductive PP, PLA and PP/PLA: T_5%_, T_50%,_ ∆T and the remaining ash (%) at 500 °C.

Yarn Type	T_5%_ (°C)	T_50%_ (°C)	∆T (°C)	Remaining Ash (%) at 500°C
PP Modified PP PEDOT:PSS coated PP	332 344 364	455 461 465	- 6 11	0.93 2.16 2.72
PLA Modified PLA PEDOT:PSS coated PLA	296 307 333	354 372 393	- 18 39	0.20 0.30 0.39
PP/PLA Modified PP/PLA PEDOT:PSS coated PP/PLA	315 317 336	386 412 430	- 26 44	1.16 2.46 4.84

∆T = temperature difference at 50% mass loss among the cross-linked samples and the neat polymers.

**Table 5:** DSC results of glass transition temperature (T_g_) and melting point (T_m_) of PDA treated and PEDOT:PSS coated PP, PLA and PP/PLA yarns.

Yarn Type	T_g_ (°C)	T_m_ (°C)
PP	-	130.92
Modified PP	-	131.98
PEDOT:PSS coated PP	-	132.61
PLA	56.44	131.55
Modified PLA	56.44	149.49
PEDOT:PSS coated PLA	56.44	149.91
PP/PLA	50.55	132.27
Modified PP/PLA	55.50	149.15
PEDOT:PSS coated PP/PLA	56.18	150.01

**Table 6:** Aging effect of PEDOT:PSS coated conductive yarns.

Conductive Yarn Type	Aging Duration (Week)	Electrical Resistance (KΩ)							
		For 1st Coating				For 2nd Coating			
		Mean	SD	Increased Electrical Resistance Every Week (%)	Total Increased Electrical Resistance (%) from Week 0–Week 5 (%)	Mean	SD	Increased Electrical Resistance (%)	Total Increased Electrical Resistance (%) from Week 0–Week 5 (%)
PP	0	131.00	3.41	-	23.43	43.00	2.08	-	20.98
	1	143.07	1.63	9.21		44.70	0.46	3.95	
	2	149.67	0.57	4.61		46.93	0.57	4.99	
	3	154.70	2.03	3.36		48.73	0.75	3.84	
	4	159.30	1.91	2.97		51.37	1.32	5.42	
	5	164.53	0.83	3.28		52.80	0.30	2.78	
PLA	0	223.00	7.76	-	26.85	122.00	5.47	-	23.00
	1	251.10	1.11	12.60		134.13	1.05	9.94	
	2	262.40	2.80	4.50		139.40	1.20	3.92	
	3	269.10	1.74	2.55		143.33	0.86	2.82	
	4	279.10	2.16	3.72		148.50	0.86	3.61	
	5	288.80	2.35	3.48		152.53	0.83	2.71	
PP/PLA	0	181.00	3.63	-	25.45	66.00	1.23	-	21.96
	1	199.50	0.79	10.22		73.43	0.65	11.26	
	2	210.50	1.12	5.51		75.67	0.57	3.05	
	3	215.33	0.96	2.29		78.50	0.87	3.74	
	4	223.40	0.79	3.75		80.80	0.36	2.93	
	5	231.63	0.42	3.68		81.63	0.87	1.03	
